# Clinical Variables as Predictors of First Relapse in Pediatric Crohn’s Disease

**DOI:** 10.7759/cureus.4980

**Published:** 2019-06-24

**Authors:** Nageshwar Chauhan, Hamza H Khan, Sanjay Kumar, Hernando Lyons

**Affiliations:** 1 Neonatology, Marshfield Clinic, Marshfield, USA; 2 Pediatrics, Ascension St. John Hospital, Detroit, USA; 3 Pediatric Gastroenterology, Ascension St. John Hospital, Detroit, USA

**Keywords:** pediatrics, crohns disease

## Abstract

Introduction

Crohn's disease (CD) is an immune-mediated inflammatory bowel disease (IBD) that can affect any portion of the gastrointestinal tract from the mouth to the anus. The clinical course of CD is characterized by periods of symptomatic relapse and remission. Clinical variables may identify a subset of patients with CD at risk for relapse. Identifying these patients, and early stratification-based treatment would be of utmost clinical importance in optimizing the management and is likely to improve long-term disease outcome. In pediatric-onset IBD there is a paucity of data for predicting clinical behavior and results are conflicting. With this background, we hypothesized that routinely measured clinical variables at the time of diagnosis would predict relapse in patients with CD, and sought to investigate the clinical predictors of relapse present at the time of diagnosis in our patient population. We further compared differences in clinical variables and laboratory values for patients who relapsed early, compared with those who relapsed late.

Methods

We conducted a retrospective chart review of patients diagnosed with CD by clinical, radiological, endoscopic and histological criteria at St. John Providence Children’s Hospital pediatric GI clinic between 01/2006 and 12/2014. Patients were followed until they had their first relapse or for three years from diagnosis, whichever was earlier. Variables studied included demographic factors (age, gender, race, BMI, BMI percentiles and family history of IBD), presenting symptoms (blood in stools, nocturnal stools, fever, and extra-intestinal manifestations), phenotypic characteristics (using Montreal classification), and laboratory data [white blood cell (WBC) count, hemoglobin, hematocrit, platelet count, erythrocyte sedimentation rate (ESR), and C-reactive protein (CRP)].

Results

Twenty-nine patients were included in the study. One was lost to follow up, and 28 were included in the analyses. The relapse rate was 50% at three years, and 32% patients relapsed within one year of diagnosis. Low BMI percentile at diagnosis (41.5 ± 28.8 vs. 18.0 ± 20.3; p-value 0.03) was a predictor of relapse. Comparing early relapse to those who relapsed late, there were no statistically significant differences between the two groups.

Conclusions

Low BMI percentile at presentation was associated with increased risk of relapse, suggesting that routinely measured clinical variables may have role in predicting first relapse in this patient population. There was no significant difference in the variable comparing patients who relapsed early vs. those who relapsed late. Future prospective studies with larger sample sizes need to be done to predict relapse.

## Introduction

Inflammatory bowel disease (IBD) is a group of chronic gastrointestinal disorders that result in autoimmune damage to a patient’s gastrointestinal (GI) tract. It is comprised of two major disorders: Ulcerative colitis (UC) and Crohn's disease (CD). UC affects the colon, whereas CD can involve any component of the GI tract from the oral cavity to the anus. IBD can affect any age group, but the incidence is increasing in children and young adults [1].

CD is thought to be a disorder likely related to an aberrant immunological response to intestinal microbiota in genetically-susceptible individuals [2]. Approximately 20 to 25% of all cases are diagnosed in children younger than age 18 years [2,3]. Most children and adolescents with CD suffer more severe, more extensive, and more complicated disease than most adults, in addition to developing unique complications including growth failure and delayed puberty [4]. Patients may be classified by age, disease location, and disease behavior, by the "Montreal" classification system [5].

The course of CD varies substantially between individuals, ranging from mild disease requiring minimal treatment, to a severe, treatment-resistant course. The treatment goal in CD is to induce and maintain remission by effective suppression of gut inflammation. Despite exhaustive studies to determine predictors of disease behavior, gastroenterologists are unable to propose an individualized treatment strategy due to the inability to predict the time of likely relapse, if present [6]. Hence, current treatment strategies, both in adults and children, follow a so-called ‘step-up’, i.e., a ‘treat-to symptom’ approach that involves a standardized, incremental treatment response according to symptoms [7]. As a consequence, a large proportion of patients either receive unnecessarily potent medications risking potentially life-threatening side effects, while others are being treated inadequately (i.e., “under treated”). Moreover, increasing evidence suggests that early stratified treatment of risk groups is likely to improve long-term disease outcome [8]. Thus, detection of such inflammation and identification of patients at high risk of relapse are of great clinical significance especially when choosing therapeutic agents and treatment strategies.

Routinely measured variables have been suggested as markers of relapse in patients with CD. Potential advantages offered by these variables in risk stratification include time efficiency, cost effectiveness and minimal variability in results when measured in different settings [9]. In adult studies, variables at diagnosis associated with aggressive CD (defined as having a high relapse rate, development of penetrating disease, need for repeat surgery, or multiple admissions for flares) have been studied and include younger age, perianal disease, involvement of upper GI, need for steroids at presentation, and smoking [10]. However, there is a paucity of literature on predictive factors associated with an initial relapse in the pediatric population. A single center retrospective study by Hojsak et al. involving 74 patients aimed at evaluating risk factors associated with the relapse rate, and the need for surgery in the first year after diagnosis in children with CD, reported no significant positive risk factor associated with relapse [11]. Another single center retrospective study aimed at predicting the risk of colectomy in pediatric-onset UC patients using UC risk score concluded that routinely measured clinical variables (i.e., WBC and Hct) at diagnosis may have a prognostic role in risk stratification as measured by increased risk of colectomy [9]. Therefore, in this study, we aim to determine if any clinical variables or laboratory values at presentation were predictors of relapse in CD, and also to determine if these values at presentation were predictors of early relapse, compared with late relapse. Based on the results of this study, hypotheses may be generated for larger, prospective studies assessing the usefulness of common clinical variables in predicting the initial relapse in this patient population.

## Materials and methods

This was a retrospective chart review of all patients with Crohn’s disease diagnosed at St. John Providence Children’s Hospital gastroenterology clinic during the period of 2006-2014. The subjects in our study had confirmed CD diagnosed on clinical, radiological, endoscopic and histologic criteria between the ages of 1 and 21. Date of diagnosis was defined as the date on which diagnosis was confirmed by endoscopy. Patients with UC and indeterminate colitis were excluded from the study. Clinical variables analyzed to predict the relapse in this population (at the time of diagnosis) included demographic parameters [age at diagnosis, gender, race, BMI percentiles (height and weight at the time of diagnosis were converted to BMI percentiles and treated as continuous variables in the analysis), time to relapse (if any), family history of IBD (defined as presence of CD or UC in the first degree relatives only)], presenting symptoms (gross blood in the stool, nocturnal stooling, and fever), phenotypic characteristics, and extraintestinal manifestations. Laboratory data collected included WBC count, hemoglobin, hematocrit, platelet count, ESR, and CRP. Patients were phenotyped using the Montreal classification. Disease activity was assessed until three years post diagnosis. Relapse was defined as an episode of increased disease activity requiring medication change or surgical intervention.

Statistical analysis

Descriptive statistics were generated to characterize the study population. Continuous variables such as age were described with the mean and standard deviation or median and interquartile range. Categorical variables were described as frequency distributions. Univariate analysis was done using chi squared analysis, Student’s t-test and analysis of variance, as appropriate. Time to relapse was assessed using Kaplan-Meier survival analysis. All data was analyzed using SPSS v. 22.0 (IBM Corp., Armonk, NY) and a p-value of 0.05 or less was considered to indicate statistical significance.

## Results

**Figure 1:**
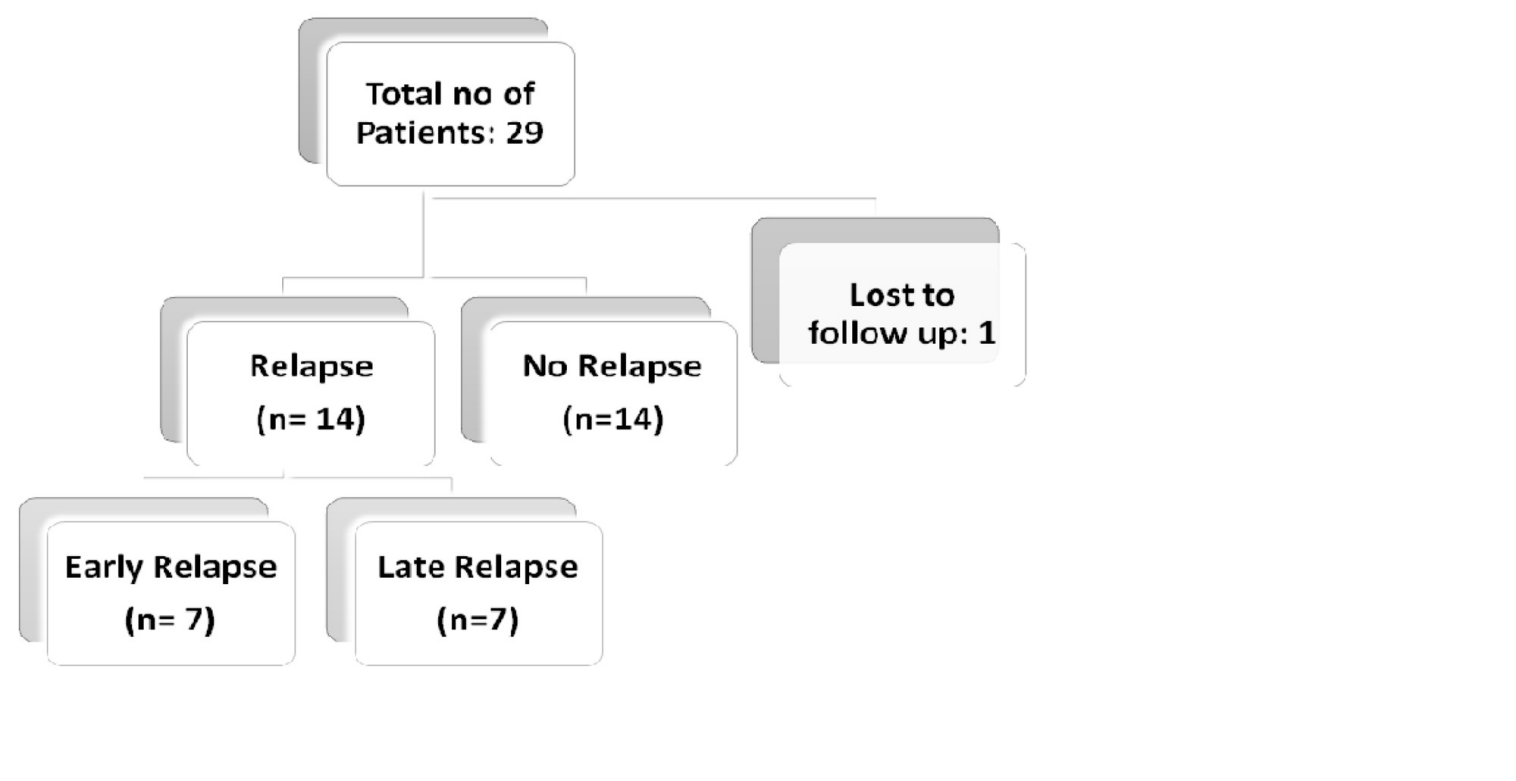
Study flow chart

Twenty-nine patients were included in the study. One was lost to follow up, and 28 were included in the analyses (Figure 1).

Mean age at presentation was 12.5 years. Table 1 summarizes the clinical and laboratory characteristics of the patient population.

**Table 1:** Clinical and lab characteristics of patient population WBC: White blood cell; Hb: Hemoglobin; Hct: Hematocrit; ESR: Erythrocyte sedimentation rate; CRP: C-reactive protein.

Variable	Mean ± SD
Age (years)	12.5 ± 3.8
BMI (kg/m^2^)	17.4 ± 3.2
BMI (%)	32.8 ± 29.2
Hb (g/dl)	11.1 ± 2.4
Hct (%)	34.1 ± 6.3
WBC (K/mcL)	10.8 ± 4.9
Platelet (K/mcL)	428.2 ± 136.2
ESR (mm/hr)	30.0 ± 18.9
CRP (mg/dL)	44.1 ± 51.4

Of all the patients, 64% were male, 68% were Caucasians, and 30% had family history of IBD. Ileo-colonic was the most common area affected, and 30% patients had extra intestinal manifestations. Table 2 summarizes the phenotypic characteristics of the patient population.

**Table 2:** Phenotypic characteristics of patient population IBD: Inflammatory bowel disease; EIM: Extraintestinal manifestations.

Variable (n)	Proportion % (n)	
Gender (28)	Male	64.3 (18)
Race (28)	Caucasian	67.9 (19)
	African-American	28.6 (8)
	Others	3.6 (1)
Family history of IBD (26)	Positive	30.8 (8)
Blood in stool (27)	Yes	40.7 (11)
Nocturnal stools (24)	Yes	4.2 (1)
Fever (24)	Yes	12.5 (3)
Location (27)	Ileal	18.5 (5)
	Colonic	3.7 (1)
	Ileo-colonic	55.6 (15)
	Ileo-colonic and upper GI	14.8 (4)
	Ileal and upper GI	3.7 (1)
	Colonic and upper GI	3.7 (1)
Behavior (27)	Inflammatory	77.8 (21)
	Stricturing	3.7 (1)
	Penetrating	3.7 (1)
	Inflammatory (p)	14.8 (4)
EIM (26)	Yes	34.5 (9)

The relapse rate was 50% at three years and 32% of patients relapsed within one year of diagnosis. Of all the patients who relapsed, the time to relapse by the first 50% of patients (t50) was seven months. Low BMI percentile at diagnosis (41.5 ± 28.8 vs. 18.0 ± 20.3; p-value 0.03) was a predictor of relapse. Table 3 summarizes the variables associated with the relapse.

**Table 3:** Continuous variables associated with relapse Hb: Hemoglobin; Hct: Hematocrit; WBC: White blood cell; ESR: Erythrocyte sedimentation rate; CRP: C-reactive protein.

Variable (n)	Relapse	No relapse	p-value
Age (years)	13.5 ± 2.8	11.5 ± 4.7	0.19
BMI (kg/m^2^)	16.6 ± 2.0	17.6 ± 3.7	0.40
BMI (%)	18.0 ± 20.3	41.5 ± 28.8	0.03
Hb (g/dl)	10.5 ± 2.2	11.4 ± 2.4	0.32
Hct (%)	32.9 ± 5.6	34.4 ± 6.7	0.54
WBC (K/mcL)	10.4 ± 4.5	10.2 ± 3.9	0.91
Platelet (K/mcL)	481.1 ± 168.0	396.9 ± 93.6	0.13
ESR (mm/hr)	31.0 ± 13.9	29.2 ± 24.6	0.74
CRP (mg/dL)	59.5 ± 70.0	31.5 ± 37.3	0.35

Comparing patients who relapsed early to those who relapsed late, there were no statistically significant differences between the two groups. Tables 4, 5 summarize the clinical, laboratory and phenotypic variables associated with relapse.

**Table 4:** Clinical and laboratory variables associated with relapse Hb: Hemoglobin; Hct: Hematocrit; WBC: White blood cell; ESR: Erythrocyte sedimentation rate; CRP: C-reactive protein.

Variable	Early Relapse	Late Relapse	p-value
Age (years)	13.4 ± 2.9	13.6 ± 2.9	0.90
BMI (kg/m^2^)	17.0 ± 2.3	16.0 ± 1.4	0.42
BMI (%)	23.3 ± 25.0	10.6 ± 8.8	0.31
Hb (g/dl)	10.4 ± 1.9	10.6 ± 2.6	0.93
Hct (%)	34.1 ± 3.5	31.6 ± 7.6	0.49
WBC (K/mcL)	11.4 ± 5.7	9.1 ± 2.3	0.38
Platelet (K/mcL)	533.7 ± 138.0	419.7 ± 191.1	0.24
ESR (mm/hr)	35.0 ± 12.9	27.8 ± 15.6	0.40
CRP (mg/dL)	85.5 ± 73.8	7.4 ± 2.0	0.23

**Table 5:** Phenotypic variables associated with relapse IBD: Inflammatory bowel disease; EIM: Extraintestinal manifestations.

Variable		ER % (n)	LR % (n)	p-value
Gender	Male	60 (6)	40 (4)	0.24
	Female	25 (1)	75 (3)	
Race	Caucasian	55.6 (5)	44.4 (4)	0.58
	African-American	40.0 (2)	60 (3)	
Family history of IBD	Positive	50 (2)	50 (2)	0.85
	Negative	55.6 (5)	44.4 (4)	
Blood in stools	Yes	50 (2)	50 (2)	0.85
	No	55.6 (5)	44.4 (4)	
Nocturnal stools	Yes	0 (0)	0 (0)	NA
	No	50 (6)	50 (6)	
Fever	Yes	50 (1)	50 (1)	1.00
	No	50 (5)	50 (5)	
Location	Ileal	50 (1)	50 (1)	NA
	Colonic	0 (0)	0 (0)	
	Ileo-colonic	66.7 (6)	33.3 (3)	
	Ileo-colonic and upper GI	0 (0)	100 (2)	
Behavior	Inflammatory	45.5 (5)	54.5 (6)	NA
	Stricturing	100 (1)	0 (0)	
	Inflammatory (p)	100 (1)	0 (0)	
EIM	Yes	60 (3)	40 (2)	0.92
	No	57.1 (4)	42.9 (3)	

## Discussion

Although the clinical predictors of relapse of CD have been extensively studied in the adult population, the literature is deficient regarding the clinical predictors in pediatric population. This retrospective study was conducted in pediatric patients who had presented to our tertiary community hospital. The overall relapse rate at one year in our study was 32%, which was lower than 48% reported by another retrospective study that was aimed to evaluate risk factors associated with the relapse rate in the first year after diagnosis and the need for surgery in children with CD, conducted in Croatia [11]. This difference could probably be attributed to the patient heterogeneity, differences in management, and regional differences in disease behavior. Authors of this study also reported that variables such as age, presence of perianal disease, CRP levels, PCDAI score, SDS weight for height, and maintenance therapy (azathioprine versus mesalamine), did not influence the relapse rate in the first year after diagnosis of CD. However, exclusive enteral nutrition (EEN) was found to prolong the duration of remission in children with CD in this study.

In this retrospective study, a number of clinical, laboratory, and phenotypic variables were studied to predict the risk of relapse. The clinical and laboratory variables that were studied included age (years), BMI (kg/m^2^), BMI (%), Hb (g/dl), Hct (%), WBC (K/mcL), platelets (K/mcL), ESR (mm/hr) and CRP (mg/dL). Our study revealed that BMI percentile was associated with early relapse in children with CD. Patients with low BMI percentile at the time of diagnosis were significantly at a higher risk of relapse compared to those with a higher BMI percentile (41.5 ± 28.8 vs. 18.0 ± 20.3, p-value 0.03). However, our study revealed that there was no significant BMI percentile difference between patients who had early relapse versus patients who had late relapse. In addition, there was no significant difference revealed studying other clinical and laboratory variables that were studied with respect to patient who had relapse versus who did not had a relapse and in patients who had early relapse versus those who had late relapse.

The phenotypic variables studied in this study included gender (male vs. female), race (Caucasian vs. African-American), family history of IBD, blood in stools, nocturnal stools, associated fever, location of the disease, behavior of the disease and EIM. Our study revealed no significant difference with respect to the phenotypic variables to predict relapse in the patients.

Strengths of the study

Current data regarding the predictors of relapse of CD in the pediatric population in the literature is very deficient. Our study was conducted in the pediatric population with mean age of 12.5 ± 3.8. Moreover, all the patients included in this study had a single provide; this ensured the uniformity of diagnosis and management of the patients.

Limitations of the study

There were many limitations to our study. Firstly, this was a single centered study. Secondly, the patients included in the study constituted a small sample size. In addition, this was a retrospective study. Lastly, although uniformity was maintained by studying only patients who were followed by a single provider, but it was noted that most of the patients were on different medicines during the course of their management, which could have influenced the relapse.

## Conclusions

Low BMI percentile at presentation was associated with increased risk of relapse, suggesting that routinely measured clinical variables may have role in predicting first relapse in this patient population. There was no significant difference in the variable comparing patients who relapsed early vs. those who relapsed late. Future prospective, multi-centered studies with larger sample sizes are recommended to predict variables of relapse in CD further.
